# The HDAC Inhibitors Scriptaid and LBH589 Combined with the Oncolytic Virus Delta24-RGD Exert Enhanced Anti-Tumor Efficacy in Patient-Derived Glioblastoma Cells

**DOI:** 10.1371/journal.pone.0127058

**Published:** 2015-05-18

**Authors:** Lotte M.E. Berghauser Pont, Anne Kleijn, Jenneke J. Kloezeman, Wouter van den Bossche, Johanna K. Kaufmann, Jeroen de Vrij, Sieger Leenstra, Clemens M.F. Dirven, Martine L.M. Lamfers

**Affiliations:** 1 Department of Neurosurgery, Brain Tumor Center, Erasmus MC, Rotterdam, The Netherlands; 2 Department of Neurosurgery, Harvey Cushing Neuro-Oncology Laboratories, Brigham & Women’s Hospital and Harvard Medical School, Boston, Massachusetts, United States of America; 3 Department of Neurosurgery, Utrecht University Medical Center, Utrecht, The Netherlands; 4 Department of Neurosurgery, Elisabeth Hospital, Tilburg, The Netherlands; University Hospital of Navarra, SPAIN

## Abstract

**Background:**

A phase I/II trial for glioblastoma with the oncolytic adenovirus Delta24-RGD was recently completed. Delta24-RGD conditionally replicates in cells with a disrupted retinoblastoma-pathway and enters cells via αvβ3/5 integrins. Glioblastomas are differentially sensitive to Delta24-RGD. HDAC inhibitors (HDACi) affect integrins and share common cell death pathways with Delta24-RGD. We studied the combination treatment effects of HDACi and Delta24-RGD in patient-derived glioblastoma stem-like cells (GSC), and we determined the most effective HDACi.

**Methods:**

SAHA, Valproic Acid, Scriptaid, MS275 and LBH589 were combined with Delta24-RGD in fourteen distinct GSCs. Synergy was determined by Chou Talalay method. Viral infection and replication were assessed using luciferase and GFP encoding vectors and hexon-titration assays. Coxsackie adenovirus receptor and αvβ3 integrin levels were determined by flow cytometry. Oncolysis and mechanisms of cell death were studied by viability, caspase-3/7, LDH and LC3B/p62, phospho-p70S6K. Toxicity was studied on normal human astrocytes. MGMT promotor methylation status, TCGA classification, Rb-pathway and integrin gene expression levels were assessed as markers of responsiveness.

**Results:**

Scriptaid and LBH589 acted synergistically with Delta24-RGD in approximately 50% of the GSCs. Both drugs moderately increased αvβ3 integrin levels and viral infection in responding but not in non-responding GSCs. LBH589 moderately increased late viral gene expression, however, virus titration revealed diminished viral progeny production by both HDACi, Scriptaid augmented caspase-3/7 activity, LC3B conversion, p62 and phospho-p70S6K consumption, as well as LDH levels. LBH589 increased LDH and phospho-p70S6K consumption. Responsiveness correlated with expression of various Rb-pathway genes and integrins. Combination treatments induced limited toxicity to human astrocytes.

**Conclusion:**

LBH589 and Scriptaid combined with Delta24-RGD revealed synergistic anti-tumor activity in a subset of GSCs. Both HDACi moderately augmented viral infection and late gene expression, but slightly reduced progeny production. The drugs differentially activated multiple cell death pathways. The limited toxicity on astrocytes supports further evaluation of the proposed combination therapies.

## Introduction

Patients with the malignant brain tumor glioblastoma have a prognosis of 12–15 months despite maximum therapy.[1] More effective therapies than the current approach of surgery, radiation and temozolomide are urgently needed. One option is oncolytic virotherapy with Delta24-RGD, which has recently completed phase I/II clinical evaluation [2] and has shown promising results *in vitro* and *in vivo*.[3, 4] Delta24-RGD is a genetically modified serotype-5 adenovirus which induces several cellular responses including necrosis, autophagy and caspase-dependent apoptosis.[5, 6] Delta24-RGD conditionally replicates in tumors with a deregulated retinoblastoma (Rb) pathway, due to a 24-base pair deletion in the *E1A* gene. An arginine-glycine-aspartic acid (RGD) domain was inserted into the viral fiber knob domain, redirecting attachment to αvβ3 and αvβ5 integrins on the cell surface.[7] Despite these modifications glioblastomas are not equally susceptible to Delta24-RGD treatment.[8] Combination strategies that facilitate viral infection, replication and oncolysis are therefore necessary to improve this therapeutic option. Such a combination treatment may utilize histone deacetylase inhibitors (HDACi), which are novel anti-cancer drugs that act through inhibition of HDACs. This results in alterations in the transcription of oncogenes and tumor suppressor genes.[9, 10] These drugs also affect non-histone targets including genes involved in cell cycle regulation, apoptosis and autophagy.[9, 11] HDACi are reported to enhance oncolytic adenoviral therapy[12], however the effects of a panel of HDACi in patient-derived glioblastoma stem-like cells (GSC), a model that recapitulates the original tumor [13], have not been evaluated yet. Previous studies provide a rationale to systematically investigate the efficacy of HDACi as enhancers for Delta24-RGD in glioblastoma. In this study we compared the *in vitro* effects of the five HDACi SAHA, LBH589, Scriptaid, MS-275 and Valproic Acid (VPA) on Delta24-RGD-induced oncolysis in fourteen patient-derived GSC cultures. We identify the most effective HDACi in combination treatment in this relevant model for glioblastoma. The effects on cell viability, viral infectivity, viral replication, as well as cellular autophagy, necrosis and apoptosis are studied. The differences between responding and resistant GSCs to combination treatment are charted. Specifically, the novel agent Scriptaid and the clinically applied LBH589, activate a variety of mechanisms including apoptosis, autophagy and necrosis, and induce viral gene expression over time. These effects were associated with up-regulation of αvβ3 integrins in responding cultures, however, viral progeny production was not increased. The effects of the combination treatment were studied in normal human astrocytes and toxicity was found to be very limited.

## Materials and Methods

### Chemicals

The HDACi tested were SAHA and MS275 (Cayman chemicals, MI, USA), VPA (Sigma-Aldrich, MO, USA), LBH589 (Biovision, CA, USA), and Scriptaid (Santa Cruz Biotechnology, CA, USA). Stocks were prepared at 100mM (VPA) in sterile water and at 50mM (SAHA), 10mM (Scriptaid), 4 mM (MS275), and 200μM (LBH589) in dimethyl sulfoxide (Sigma-Aldrich) and stored at -20°C. Staurosporin was obtained from BioMol (Hamburg, Germany).

### Viruses

The construction of Delta24-RGD has been described previously.[14] The adenoviral construct has a 24-base pair deletion in the viral *E1A* gene, which disrupts the Rb-binding capacity of this protein and facilitates selective replication in cells with a dysfunctional Rb-pathway. The RGD peptide allows the virus to bind and enter the cell through cell surface integrins αvβ3/5.[15] The Delta24-RGD-GFP virus was constructed for the purpose of monitoring late viral gene expression over time and was used to evaluate the viral behavior by fluorescent imaging. The virus contains a GFP-expression cassette under control of the *E3*-promotor, and was constructed, produced, purified and titrated as described previously.[16] The replication-deficient adenoviral vector Ad.luc.RGD, kindly provided by Dr. D.T. Curiel, (University of Alabama, Birmingham, Al, USA), was used for the infectivity experiments.

### Patient-derived serum-free cultured glioblastoma stem-like cells

Fresh tumor material was obtained from surgical resection at the Department of Neurosurgery of the ErasmusMC (Rotterdam, The Netherlands). The tumors were classified as WHO grade IV (glioblastoma) by histopathological assessment. This entire study including the use of patient tumor material for the current study was approved by the institutional review board of the ErasmusMC. The patients’ written informed consent was acquired for the use of patient material for our studies. The tumors were dissociated mechanically and enzymatically as described previously.[17] Both the primary cultures and parental tumors were characterized and subtyped as described previously.[17] Fourteen patient-derived GSCs were used in the experiments and are summarized in Table 1. The cells were cultured as tumor neurospheres in serum-free DMEM/F12, supplemented with 1% penicillin/streptomycin, 2% B27, 20ng/ml bFGF, 20ng/ml EGF (Life Technologies, Paisley, UK), and 5μg/ml heparin (Sigma-Aldrich). For the experiments, the cell culture plates were coated with Cultrex (Trevigen, MD, USA) allowing attachment and monolayer formation for reproducible *in vitro* analysis. The cultures were maintained at 37°C in a humidified chamber (95% air/5% CO_2_).

**Table 1:** Overview of the patient-derived GSC cultures tested for combination treatment.

Primary GSC culture	*MGMT* methylation status	Molecular subtype	Scriptaid	LBH589	SAHA	VPA	MS275
**GS289**	UM	CLA	2.12	1.58	NR	NR	1.27
**GS245**	UM	NEU	NR	NR	NR	NR	NR
**GS160**	UM	CLA	NR	1.26	NR	1.35	1.22
**GS184**	M	CLA	1.31	NR	NR	1.48	NR
**GS224**	M	CLA	1.78	4.4	2.71	NR	1.63
**GS102**	M	NEU	1.66	2.49	1.33	NR	NR
**GS257**	UM	CLA	2.9	NR	NR	NR	NR
**GS79**	UM	CLA	NR	NR	NR	NR	1.16
**GS209**	UM	PRO	1.23	1.37			
**GS249**	M	CLA	NR	NR			
**GS274**	M	ND	NR	NR			
**GS335**	M	CLA	NR	1.22			
**GS359**	M	MES	1.28	1.39			
**GS368**	UM	CLA	1.38	NR			

Fourteen patient-derived GSC cultures were tested for the responsiveness to the five HDACi and Delta-24-RGD. The MGMT promoter methylation status and the molecular TCGA characterizations are depicted. The enhancement factors are shown for the various treatment modalities if EF >1, and if there was a significant difference of the combination treatment compared to the monotreatments (p<0.05). NR indicates non-responsiveness to the proposed combination treatment. Legends: UM = unmethylated; M = methylated; CLA = classical; NEU = neural; PRO = proneural; MES = mesenchymal; ND = not determined.

### Viability assays

Primary GSC cultures and normal human astrocytes (Sciencell, CA, USA) were seeded at 1x10^3^ cells per well in 96-well plates. The cells were treated with the HDACi SAHA, Scriptaid, LBH589, MS275, and VPA in dose-increasing concentrations concomitantly with the Delta24-RGD infection. The tested virus doses were 5, 10, 25 and 50 infectious virus particles/ cell (multiplicity of infection; MOI) to ensure a viral dose that corresponded with the IC_10_—IC_50_ value of the treatment, the optimal dosages for studying combination effects. Cell viability was assessed seven days post-treatment using the CellTiter-Glo cell viability assay (Promega, WI, USA). At least three MOIs of the virus were used for these assays and the IC_50_ values were determined by median equation.

Next, the stringent approach to determine synergy between compounds, the Chou Talalay assay[18], was used for the most effective HDACi in combination with Delta24-RGD. The combination index of the two agents was calculated by using this method. The experiments were performed in triplicate and results are shown as percentage of non-treated controls ± standard deviation. In addition, primary human astrocytes were analyzed for viability as well as cell confluence after five days of combination treatment. These results are also shown as percentage of non-treated controls ± standard deviation. Microscopic images were obtained using the IncuCyte imaging system (Essen Bioscience, Ann Arbor, MI, USA) and are presented in phase-contrast at 10X magnification.

### Gene expression analysis, TP53 mutation status, and MGMT promoter methylation status

The *TP53* mutation status was available of ten GSCs, and was derived from next-generation DNA sequencing using an AB SOLiD sequencer on the fresh frozen parental tissues of the primary GSCs. In addition, molecular subtyping was done using available mRNA expression data[17] according to the transcriptionally-defined TCGA classification.[19] The *MGMT* promoter methylation status of the patient-derived GSCs was determined as described previously. [20]The mRNA of the parental glioblastoma tissue of the GSCs was isolated with the RNeasy Mini kit (#74104, Qiagen Inc., CA, USA) The expression levels were analyzed by the HumanHT-12 v4 Expression BeadChip microarray (Illumina, CA, USA), as we have described previously.[21] The normalization and batch effect removal was done as described previously by using Partek software, version 6.6 (Partek Inc., St. Louis, MO, USA). The expression data was used to subtype the tumors to the transcriptionally-defined glioblastoma classification of the TCGA.[22] We looked for patterns between responsiveness and molecular subtype. The average expression levels of specific genes of responders and non-responders to treatment were compared and the fold change was calculated including the p-value by Student’s T-test. The KEGG pathways were used for identifying Rb-pathway genes.

### Adenoviral infection experiments

The patient-derived GSC cultures GS102, GS224, GS289, GS79, GS245 and GS249 were seeded at 5x10^3^ cells per well in 96-well plates. After 24 hours the cells were treated with the concentrations of Scriptaid (1.5μM) and LBH589 (15nM) at which synergy with Delta24-RGD was measured. Ad.Luc.RGD was applied to the cells at MOI25. After 24 hours the supernatant was removed and the cells were lysed using 0.9% Triton-X100. The plates were frozen and infectivity was quantified by using the Luciferase Assay System (Promega) according to manufacturer’s instructions. Luminescence was measured with a Tecan Infinite Reader (Tecan Group Ltd., Mannedorf, Switzerland). The experiments were performed in triplicate and the results are shown as mean absolute luciferase signal ± standard deviations.

### Flow cytometric analysis of integrin and CAR expression

The cells of GS102, GS289, GS79 and GS245 were seeded in 6-well plates at 5x10^4^ cells per well and treated with Scriptaid (0.5 and 1.5μM) or with LBH589 (5nM, 15nM, or 45nM). The cells were harvested after 6 and 24 hours, were washed and incubated for 15 minutes with FACS buffer (PBS/0.25% bovine serum albumin (BSA)/0.05% NaN3/0.5mM Ethylene-di-amine-tetra-acetic acid (EDTA)/2% human serum) containing primary antibodies against integrin αvβ3 (mouse anti-CD51/CD61, 1:50, Abcam) and CAR (rabbit anti-CAR, H-300, 1:50, Santa Cruz). After washing with FACS buffer, the cells were incubated with secondary antibodies Alexa-488 anti-rabbit and PE-anti mouse (Life Technologies). After staining and fixing with BD FACS lysing buffer (BD Biosciences, San Jose, California) a minimum of 3x10^4^ events were acquired on a FACS Canto II (Becton Dickinson, San Jose, California). The analysis of the flow cytometry data was done using Infinicyt software (Cytognos, Salamanca, Spain). Debris and doublets were removed using FSC-H and FSC-A after which expression was plotted for the remaining events.

### Monitoring late viral gene expression by fluorescence imaging

The cells of the GSCs GS102, GS224 and GS289 were at 2.5x10^3^ cells per well in a 96-wells plate. After 24 hours the cells were treated with LBH589 or Scriptaid and the Delta24-RGD-GFP virus. Fluorescence intensity was detected by the IncuCyte imaging system for120 hours post-infection, and was quantified by the IncuCyte software as intensity per mm^2^ and by ImageJ fluorescent count per well. The experiments were performed in triplicate and are presented ± standard deviations.

### Viral titration assay

The cells of GS102, GS224, GS79 and GS245 were seeded at 5x10^4^ cells per well. After 24 hours the cells were treated with Delta24-RGD in combination with Scriptaid or LBH589. After 48 and 96 hours the cells and supernatants were collected. The samples underwent three freeze-thaw cycles and were centrifuged at 1.5x10^**3**^ rpm for 3 minutes to remove cell debris. Supernatants were added in serial dilutions in a titration setting to A549 lung adenocarcinoma cells (ATCC, VA, USA), which were seeded at 1x10^3^ cells per well. After 48 hours the cells were fixed with ice-cold methanol, washed in in PBS/0,05% Tween-20 (Sigma-Aldrich) and stained with primary mouse anti-hexon antibody in PBS/1% BSA from the Adeno-X Rapid Titer Kit (#632250, Clontech, CA, USA). After counting the hexon plaques under a microscope, the viral titers were calculated. The results are displayed as the mean viral titers of triplicates. Significant different titers were considered as such in case of p<0.05.

### Caspase-3/7 apoptosis assay

The cells of GS102, GS224 and GS289 were seeded at 5x10^3^ cells per well in a 96-well plate and incubated with the concentrations of Scriptaid (1.5μM) and LBH589 (15nM) at which synergy was detected. Virus was added at MOI15 or MOI25 followed by the addition of 5μM of reagent of the CellPlayer 96-Well Kinetic Caspase-3/7 Apoptosis Assay (Essen Bioscience). The plates were placed in the IncuCyte imaging system at 37°C in a humidified 95% air/5% CO_2_ chamber. Three images/well were taken every two hours in both phase-contrast and fluorescence for 60 hours. Caspase-3/7 activity is presented as counts per well. Experiments were performed in triplicate and means are plotted as percentages of non-treated controls ± standard deviations.

### Western blot analysis

Western blot analysis was performed on protein extracts from the cells of the primary GSC culture GS102, treated with LBH589 and Scriptaid in combination with Delta24-RGD at the synergistic concentrations. The cells were seeded and treated with HDACi/Delta24-RGD. At 72 hours after treatment cells were harvested, washed with PBS and lysed with RIPA lysis buffer (Sigma-Aldrich). Protein concentrations were measured using the BCA Protein Assay Reagent Kit (Roche, Basel, Switzerland). Protein separation was performed on pre-casted 4–15% sodium dodecyl sulphate-polyacrylamide gel electrophoresis (SDS-PAGE) gels (Bio-Rad, CA, USA) and blotted onto a polyvinylidene fluoride membrane (Immobilon-P, Millipore). Blocking was carried out with 5% non-fatty milk in Tris-Buffered Saline-Tween 20 (TBS-T) for 45 min at room temperature. Membranes were incubated overnight with primary antibodies against β-actin (1:5,000, Millipore, MA, USA), LC3BI/II, (1: 500, Cell Signalling), p62 (1:500, # ab56416, Abcam, Cambridge, UK), phospho-p70S6K (1:500, Cell Signalling) in 5% BSA/TBS-T. The membranes were washed with TBS-T and incubated with secondary antibody for 1.5 hour at room temperature (anti-rabbit-HRP, anti-mouse-HRP; 1:2000, Dako Denmark A/S, Glostrup, Denmark). Staining of β-actin was used as protein loading control. Proteins were visualized by the Pierce ECL kit (Roche), ChemiDoc MP (Bio-Rad) and ImageLab version 4.1 (Bio-Rad).

### Lactate dehydrogenase assay

The cells of GS102, GS224 and GS289 were seeded at 1.5x10^3^ cells per well in a 96-well plate and incubated with two concentrations of Scriptaid and LBH589 and Delta24-RGD. The cells were incubated for five days after which the amount of LDH in the supernatant was determined using the CytoTox-One assay (Promega) according to the manufacturer’s protocol. The fluorescence was measured in a Tecan Reader. The results are presented as LDH levels per viable unit as calculated from the percentage viability of non-treated controls. The experiments were performed in triplicate. The means are plotted ± standard deviations.

### Statistical analysis

Patient-derived GSC cultures were classified as sensitive to therapy (responder) when the enhancement [23] was > 1, and the combination treatment was significantly better than either monotherapy (p<0.05). The samples that did not meet these criteria were defined as resistant to combination treatment (non-responders). The experiments were performed in triplicate and the results are presented as mean percentage of the non-treated controls ± standard deviation. Student’s T-test was used to determine differences, which were considered significant if p<0.05. The time-based assays were analyzed by comparing single treatment to combination treatment cells by one-way ANOVA and a Tukey’s Post-Test.

## Results

### HDAC inhibitors enhance Delta24-RGD oncolysis in a subset of GSC cultures

**Fig 1:**
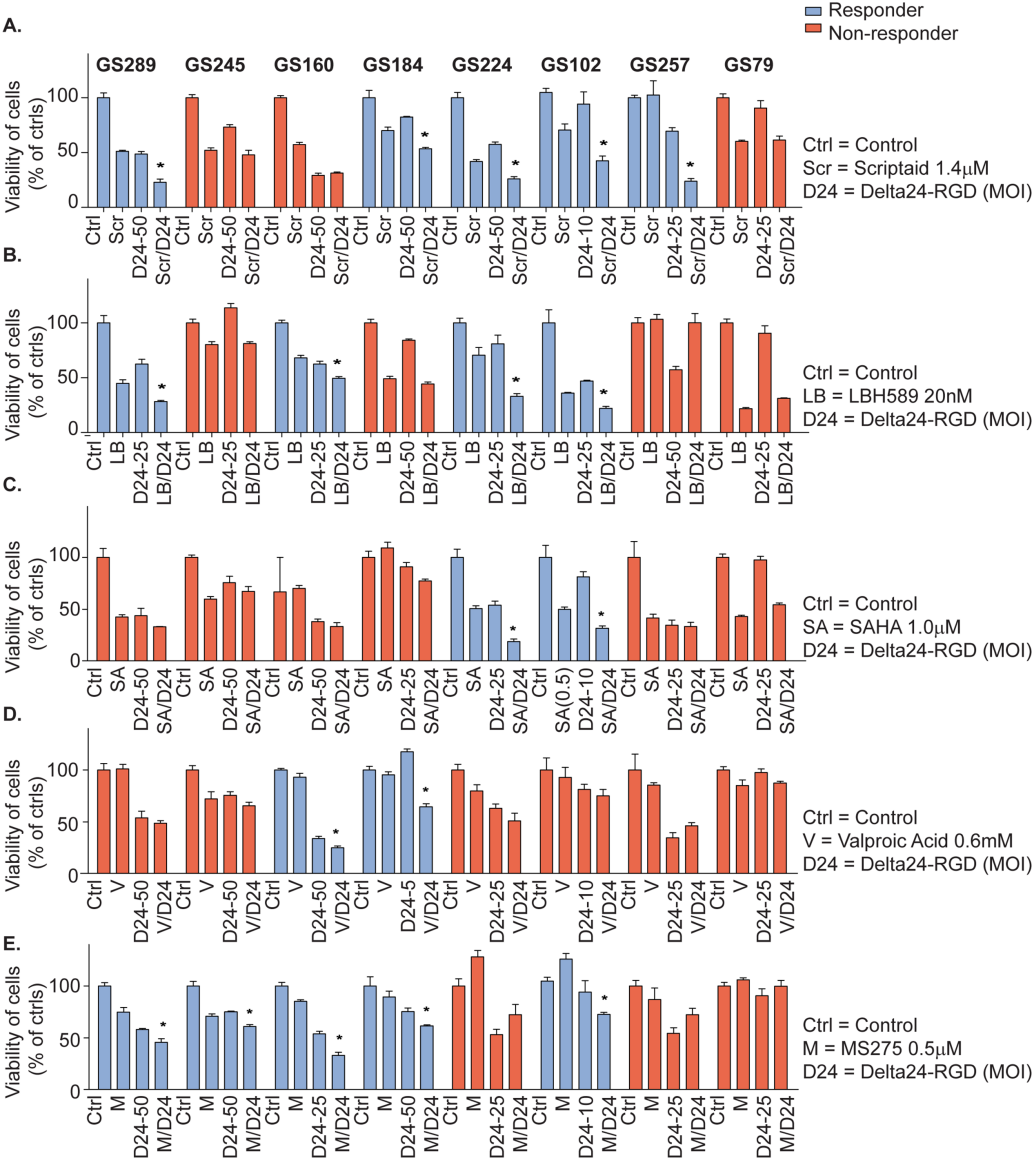
Delta24-RGD combined with various HDACi in patient-derived GSC cultures. (A-E) The eight patient-derived GSC cultures that were treated according to a simultaneous treatment schedules of HDACi and Delta24-RGD at indicated concentrations and MOIs, respectively. The treatments were added concomitantly to the cells. Results are shown for one dose of drug and one dose of oncolytic virus, for Scriptaid (A), LBH589 (B), SAHA(C), VPA (D) and MS275 (E). Response was defined as a reduction in viability, which was significantly different from both mono-treatments (p<0.05), and is indicated in the graph by the blue bars (responders). If not meeting these criteria, the culture was defined as resistant (red bars). The results are displayed by the mean viability % ± standard deviations of triplicates. *Indicates significance of combination treatment compared to HDACi or Delta24-RGD alone, p<0.05.

The combination assays with the five HDACi Scriptaid, LBH589, MS275, SAHA and VPA combined with Delta24-RGD were performed on eight GSCs. Delta24-RGD and drug treatment were applied simultaneously. At least two drug concentrations with two virus MOIs were tested per culture. The combinations of Scriptaid and LBH589 with Delta24-RGD were most effective. Scriptaid and LBH589 both enhanced the oncolytic activity of Delta24-RGD in 5/8 and 4/8 (Fig 1) of the tested GSCs with enhancement factors ranging from 1.2–2.9 for Scriptaid and 1.2–4.4 for LBH589, respectively (Table 1). SAHA and VPA were less effective than MS275, LBH589 and Scriptaid in enhancing viral oncolysis. In 2/8 of the patient-derived cultures SAHA/Delta24-RGD had additional effects (Fig 1), enhancing 1.3-fold and 2.7-fold (Table 1). Furthermore, 2/8 GSCs responded to VPA/Delta24-RGD treatment (Fig 1) with enhancing effects of 1.4 and 1.5 (Table 1). The MS275/Delta24-RGD combination showed benefit in 5/8 GSCs (Fig 1) with enhancing factors of 1.2–1.6 (Table 1). Based on the enhancement factors as well as the percentage of GSCs that were sensitized by the drug, Scriptaid and LBH589 were selected for further evaluation in combination with Delta24-RGD in six additional GSCs. Scriptaid and LBH589 showed combined additive effects with Delta24-RGD effects in 8/14 and 9/14 of the tested GSCs, respectively (Table 1, S1 Fig).

### Correlation of molecular characteristics with HDACi/Delta24-RGD treatment response

We evaluated whether specific molecular characteristics of the various GSCs were associated with HDACi/Delta24-RGD response (S1 Table). A variety of molecular parameters were evaluated as markers for response, including the *TP53* status, the tumors’ HDAC, Rb-pathway and integrin gene expression levels, *MGMT* promoter methylation status (Table 1) and the molecular subtyping classification according to the TCGA (Table 1). None of the markers except for three Rb-pathway genes and integrin expression levels, showed a significant relationship with responses to combination treatments at the p>0.01 level. For LBH589/Delta24-RGD, expression of the Rb-pathway genes mitotic arrest deficient-like 2 (*MAD2L2*, FC = 1.23, p = 0.001), E2F transcription factor 2 (*E2F2*, FC = 1.63, p = 0.007) and *CDC16* (FC = -0.44, p = 0.008) were related to response. Integrin expression levels of αv (*ITGAV*) were related to Scriptaid/Delta24-RGD response (FC = 0.48, p = 0.003). However, these relationships showed small fold-changes. In conclusion, weak correlations were found between response to combination treatment and Rb-pathway gene and integrin expression levels.

### LBH589 and Scriptaid synergize dose-dependently with Delta24-RGD

**Fig 2:**
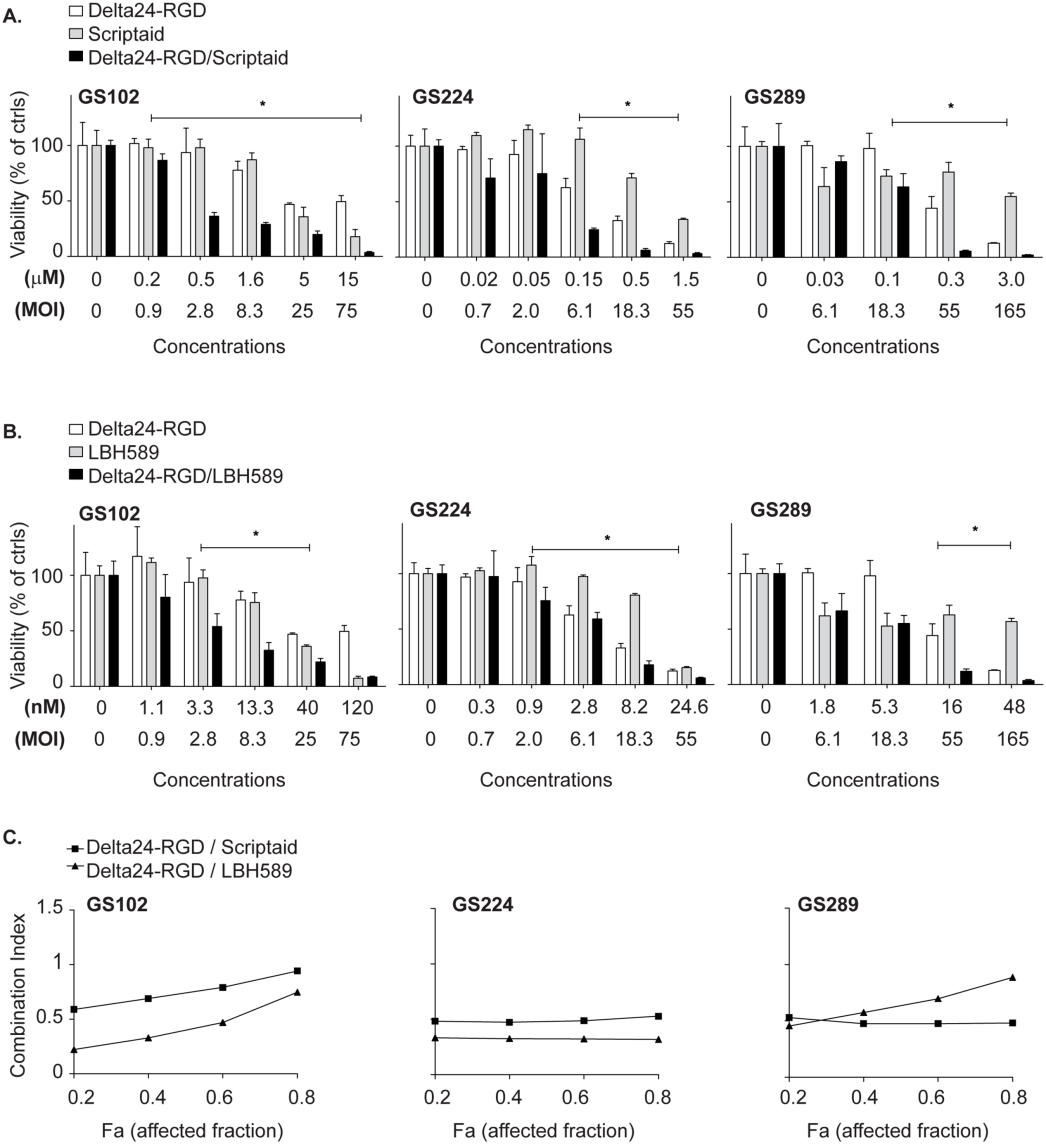
Scriptaid and LBH589 synergize with Delta24-RGD oncolysis in patient-derived GSC cultures. (A) Chou Talalay assays were performed for Scriptaid (μM) and Delta24-RGD (MOI) to determine synergy in the GSCs GS102, GS224 and GS289. The treatments were added concomitantly to the cells. The results were depicted as viable fraction (0–100) compared to non-treated controls ± standard deviation for three replicates. *Indicates significant difference between combination and single agent treatments alone at p<0.05 level. (B) Chou Talalay assays were performed for LBH589 (nM) and Delta24-RGD (MOI) to determine synergy in the responsive GSCs GS102, GS224 and GS289. The treatments were added concomitantly to the cells. The results were depicted as viable fraction compared to non-treated controls ± standard deviation for three replicates. *Indicates significant difference between combination and single agent treatments alone at p<0.05 level. (C) The combination indices (CI) for both drugs and Delta24-RGD were calculated and shown in the same graph per GSC. A CI of < 1 indicates synergy between the two agents.

Combination assays using the Chou Talalay method [18] were performed on three responsive GSCs, GS102, GS224 and GS289, to evaluate synergy between the HDACi Scriptaid or LBH589 and Delta24-RGD. First the IC_50_ values of Scriptaid, LBH589 and Delta24-RGD were determined by median-equation calculation and subsequently the combination assays were performed. The results revealed that both Scriptaid and LBH589 enhanced the oncolytic effects of Delta24-RGD in a synergistic manner (Fig 2) with combination indices below 1 (Fig 2). Scriptaid significantly sensitized for Delta24-RGD in the concentration range 0.2–14μM. LBH589 significantly sensitized for Delta24-RGD in the concentration range 1.8–48nM. Thus, the HDACi Scriptaid and LBH589 both acted synergistically with Delta24-RGD in all three responsive GSCs.

### LBH589 and Scriptaid increase RGD-mediated adenovirus infection in GSC cultures

**Fig 3:**
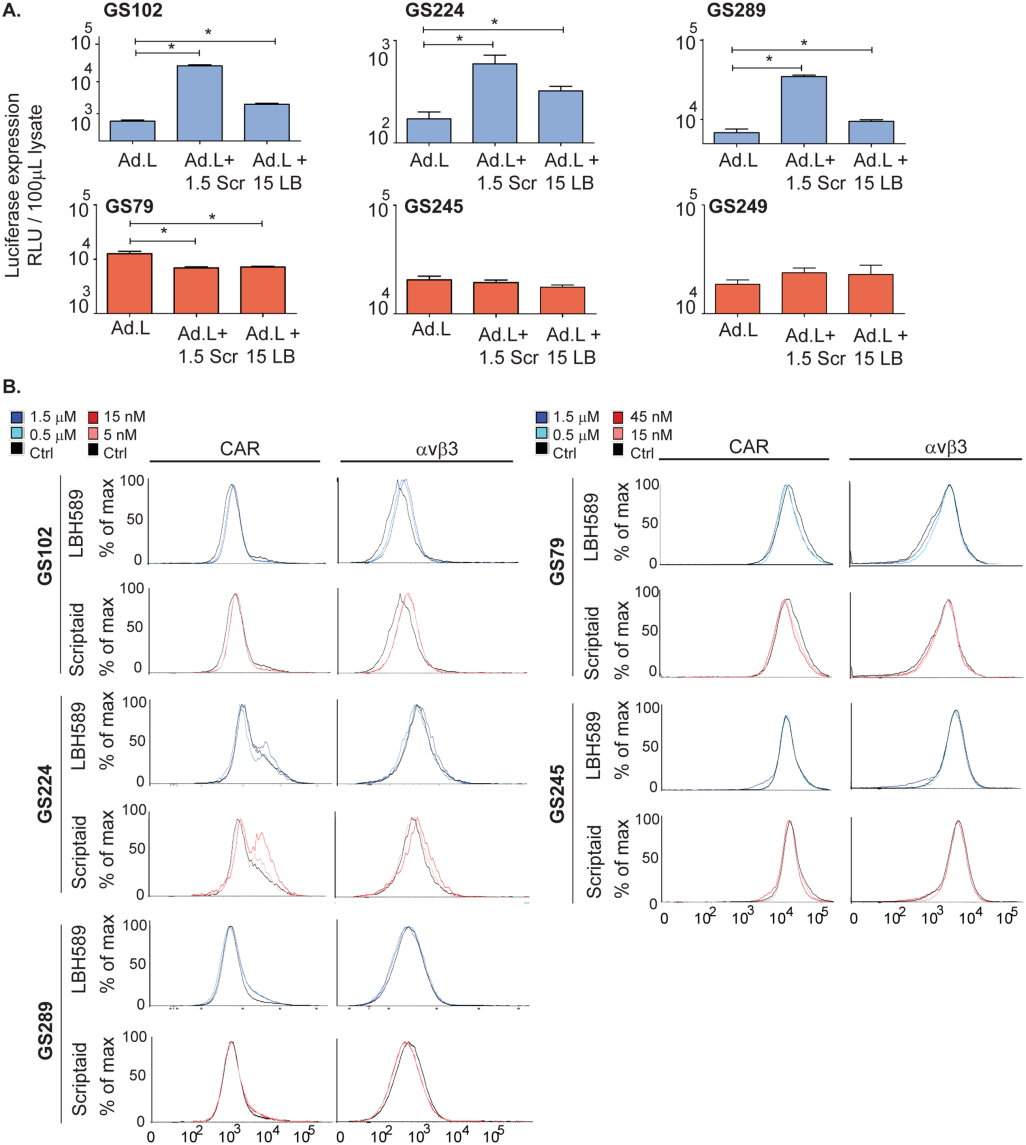
Increased Ad.luc.RGD infection and integrin levels by HDACi treatment in responding patient-derived GSC cultures. (A) Responsive GSC cultures (blue, GS102, GS224 and GS289) and resistant cultures (red, GS79, GS245 and GS249) were treated with LBH589 (LBH, 15nM) or Scriptaid (Scr, 1.5μM) and concomitantly infected with the luciferase expressing vector Ad.luc.RGD. Luciferase expression was measured after 24 hours post-infection. The results are presented as relative luciferase units (RLU)/100 μL lysate. *Indicates significance of combination treatment compared to virus alone (p<0.05). (B) The cells of three responders (left, GS102, GS224 and GS289) and two non-responders (right, GS79 and GS245) were treated with LBH589 (blue) and Scriptaid (red) as indicated. After 6 hours (S3 Fig) and 24 hours (shown) the cells were harvested and the integrin αvβ3 (left) and CAR (right) levels were determined by flow cytometry analysis.

To determine whether the combination effects of Delta24-RGD and HDACi were associated with enhanced viral infection, six different GSCs were infected with the replication-deficient vector Ad.luc.RGD in combination with Scriptaid and LBH589. Three of these GSCs were responsive to HDACi/Delta24-RGD treatment (GS102, GS224 and GS289) and three cultures were resistant (GS79, GS245 and GS359). The concentrations used were in the range at which synergy was detected in the Chou Talalay assays, namely 15nM for LBH589 and 1.5μM for Scriptaid. Luciferase expression was determined as a measure of infection efficiency (Fig 3). The results show that LBH589 significantly enhanced the Ad.luc-RGD-mediated luciferase expression (p<0.05) in all three responders, GS102 (p<0.01), GS224 (p = 0.03) and GS289 (p = 0.04, Fig 3). Similarly, Ad.luc.RGD combined with Scriptaid also resulted in significantly higher luciferase levels compared to Ad.luc.RGD alone in GS102 (p<0.01), GS224 (p = 0.03) and GS289 (p<0.01). The two HDACi did not enhance the Ad.luc.RGD-mediated luciferase expression in the resistant glioblastoma cultures. In fact, for GS79 even a decrease of infection efficacy was observed (p<0.05). To summarize, the HDACi increased viral infection in the three responsive GSCs but not in the three resistant GSCs.

### LBH589 and Scriptaid moderately increase αvβ3 integrin levels in responsive cultures

As a next step, we evaluated whether the increased infection efficacy in the HDACi-treated responsive cultures was related to increased levels of the Delta24-RGD binding sites. Therefore flow cytometric analysis of surface expression of the coxsackie adenovirus receptor (CAR) and αvβ3 integrins was performed on three responsive GSCs, GS102, GS224 and GS289, as well as two resistant cultures, GS79 and GS245 (Fig 3). The cells were treated with two concentrations of HDACi as in previous experiments. At 6 hours post-treatment, LBH589 and Scriptaid increased CAR and integrin levels in the responsive GS102. In GS289, LBH589 did not affect cell surface receptor levels and only the high concentration of Scriptaid increased CAR and integrin levels (S2 Fig). At 24 hours the αvβ3 levels were elevated in GS102 and GS224 by Scriptaid and in GS102 also by LBH589. CAR levels had returned to baseline levels in GS102. On the contrary, in GS289 and in the resistant GSCs, GS79 and GS245, no alterations were observed in αvβ3 or CAR levels. To summarize, HDACi primarily increased integrin levels in responders and not in non-responders. The results suggest that up-regulation of the important cell surface receptor for Delta24-RGD, namely αvβ3 integrin, plays a role in the increased cytotoxicity of Delta24-RGD/HDACi treatment.

### LBH589 and Scriptaid increase late viral expression but decrease progeny production

**Fig 4:**
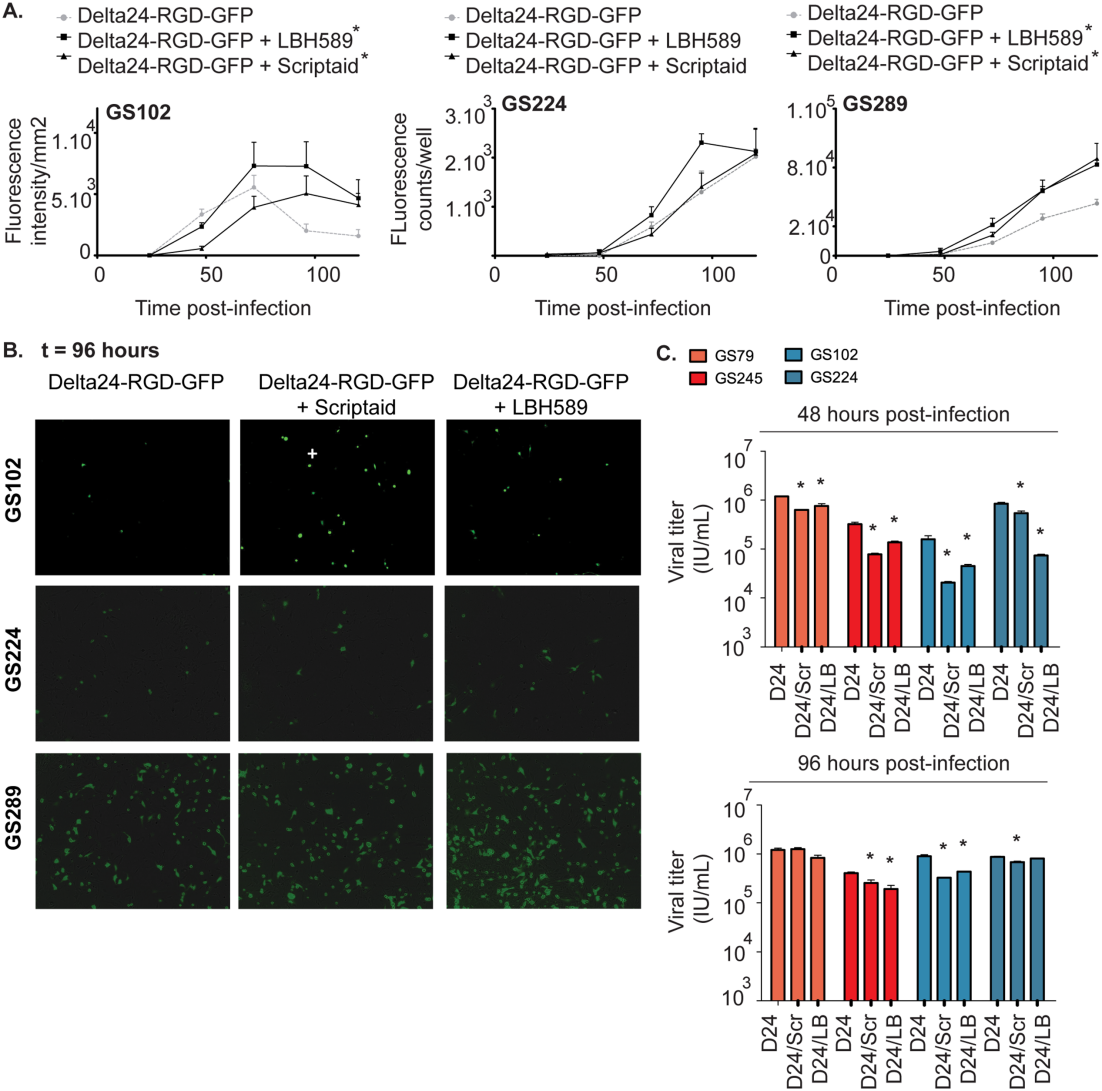
The effects of HDACi on viral transgene expression, replication and production. (A) GS 102, GS224 and GS289 cells were concomitantly treated with LBH589 or Scriptaid and Delta24-RGD-GFP virus. In a time-lapse, fluorescence intensity per mm^2^ and counts per well were measured and analyzed using the IncuCyte system and Image J, respectively. The results were plotted graphically as means ± standard deviation. *Indicates significance of the combination treatments compared to Delta24-RGD alone (p<0.05). (B) Representative fluorescence images acquired by the IncuCyte system at 96 hours are shown of GS102, GS224 and GS289 (magnification 10X). (C) Cells of the responsive GSCs GS102 and GS224 (blue), and cells of the non-responsive GSC cultures GS79 and GS245 (red) were concomitantly treated with Delta24-RGD and LBH589 or Scriptaid. The viral titers in the cell extracts that were obtained at 48h and 96h post-treatment are shown. *Indicates significant difference in the viral titers of Delta24-RGD alone compared to the combination treatments with HDACi and Delta24-RGD (p<0.05).

We studied the effects of HDACi on the pattern of late viral gene expression using the GFP-encoding variant of the virus, Delta24-RGD-GFP, which has the GFP under the control of the adenoviral E3 promoter. This virus allows *in vitro* real-time monitoring of late viral gene expression as a marker for viral replication (Fig 4). The results in responders GS102 and GS289, show that combination treatment with LBH589 or Scriptaid increased the GFP expression compared to Delta24-RGD-GFP alone (p<0.05). In GS102, Scriptaid led to a delayed onset of GFP intensity increase compared to Delta24-RGD-GFP and increased the GFP expression between 100–150 hours post-infection (p<0.05). There was no significant increase in GFP expression by the HDACi in GS224.

To evaluate whether increased levels of late viral gene expression translate to increased viral progeny production, we performed viral titration experiments for the Delta24-RGD/HDACi combination treatments on two responder (GS102 and GS224) and two non-responder (GS79 and GS245) GSCs (Fig 4). Analysis of lysates of treated cells did not reveal an increase in titers of infectious viral particles at 48 or 96 hours post-treatment, but a decrease in both responsive and non-responsive cultures (p<0.05).

### Both HDACi enhance necrosis, whereas Scriptaid also enhances apoptosis and autophagy

**Fig 5:**
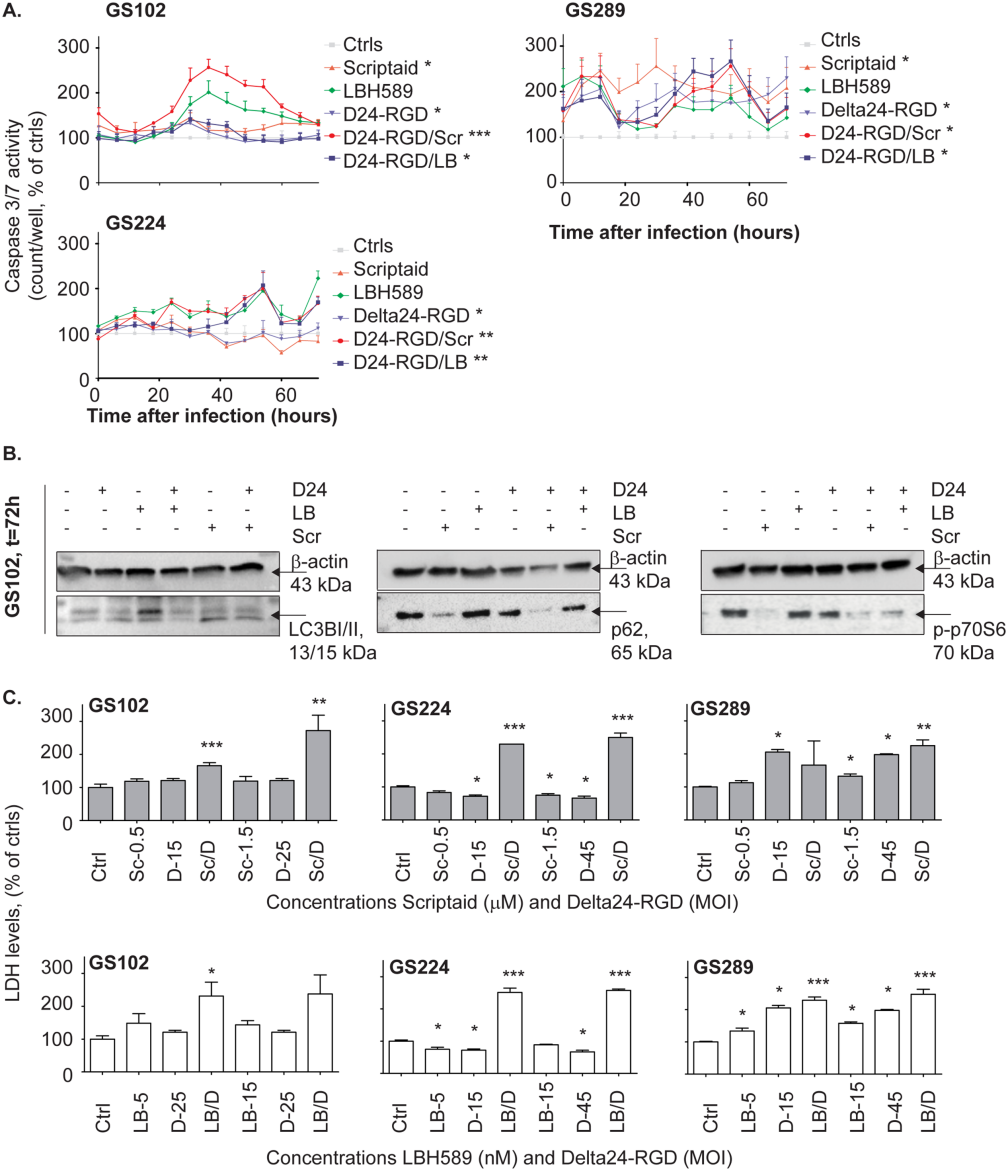
Affected cell death mechanisms in HDACi/Delta24-RGD treatment. (A) The cells of GS102, GS224 and GS289 cells were concomitantly treated with HDACi and Delta24-RGD. Caspase-3/7 activity was measured using caspase-3/7 apoptosis assay and the IncuCyte system and counts/well are shown *Indicates significance at p<0.05 for single treatments compared to non-treated controls ± standard deviation; and **/*** Indicates significance at p<0.05 for the combination treatments compared to controls (**) and the two single agent treatments (***). (B) The cells of GS102 were concomitantly treated with HDACi and Delta24-RGD. After 72 hours, the cells were harvested and LC3BI/II, p62 and phospho-p70SK levels were determined by Western blot analysis. Beta-actin was detected as a loading control. (C) The cells of GS102, GS224 and GS289 cells were concomitantly treated with Scriptaid (upper panel, grey) or LBH589 (lower panel, white) and Delta24-RGD. The LDH levels were determined using the CytoTox-One Assay reagent at five days post-treatment. Fluorescence was measured with the Tecan reader. The LDH levels are presented as percentage of non-treated controls ± standard deviation, corrected for viability. * Indicates significance at p<0.05 for the combination treatments compared to controls (**) and the two single agent treatments(***).

Next, we assessed whether enhanced viral oncolytic activity by HDACi could be attributed to activation of the cell death-associated mechanisms apoptosis, autophagy or necrosis, which are all known to be relevant in oncolytic viral therapy. Fig 5 shows the caspase 3/7 activity, a marker for apoptosis, for the three responsive cultures GS102, GS289 and GS224. Caspase-3/7 activity was increased by Scriptaid in two of three cultures. Delta24-RGD induced caspase activity in all three GSCs. The caspase activity was further augmented by co-incubation with Scriptaid (p<0.05) in GS102. In contrast, LBH589 did not effectively alter virus-induced caspase-3/7 activity.

For detection of autophagy, Western blotting was performed to visualize the conversion of the LC3BI to the LC3BII protein as well the consumption of the p62 protein (sequestosome-1) and phospho-p70S6kinase (Fig 5). In GS102 cells Scriptaid as well as Delta24-RGD/Scriptaid increased the LC3BII levels at 72 hours post-treatment. This increase in conversion was less evident for the single and combination treatment with LBH589. In support of the effects of Scriptaid on autophagy induction, both p62 and phospho-p70S6K levels were decreased by Scriptaid in both mono and combination therapies (Fig 5), whereas p62 was consumed more in the combination modality.

The cell death marker LDH was studied to evaluate whether necrotic response occurred after combination treatment. Five days post-treatment, the LDH levels were elevated in all three responsive GSCs, after combination treatment with Scriptaid/Delta24-RGD (Fig 5). This also occurred after combination treatment of LBH589/Delta24-RGD in GS224 and GS289. The degree of LDH increase varied between the different GSC cultures. In conclusion, the combination effects of HDACi/Delta24-RGD appear based, at least partly, on the induction of caspase-3/7-mediated apoptosis, and of autophagic and necrotic cell death.

### Primary human astrocytes are insensitive to HDACi and Delta24-RGD

**Fig 6:**
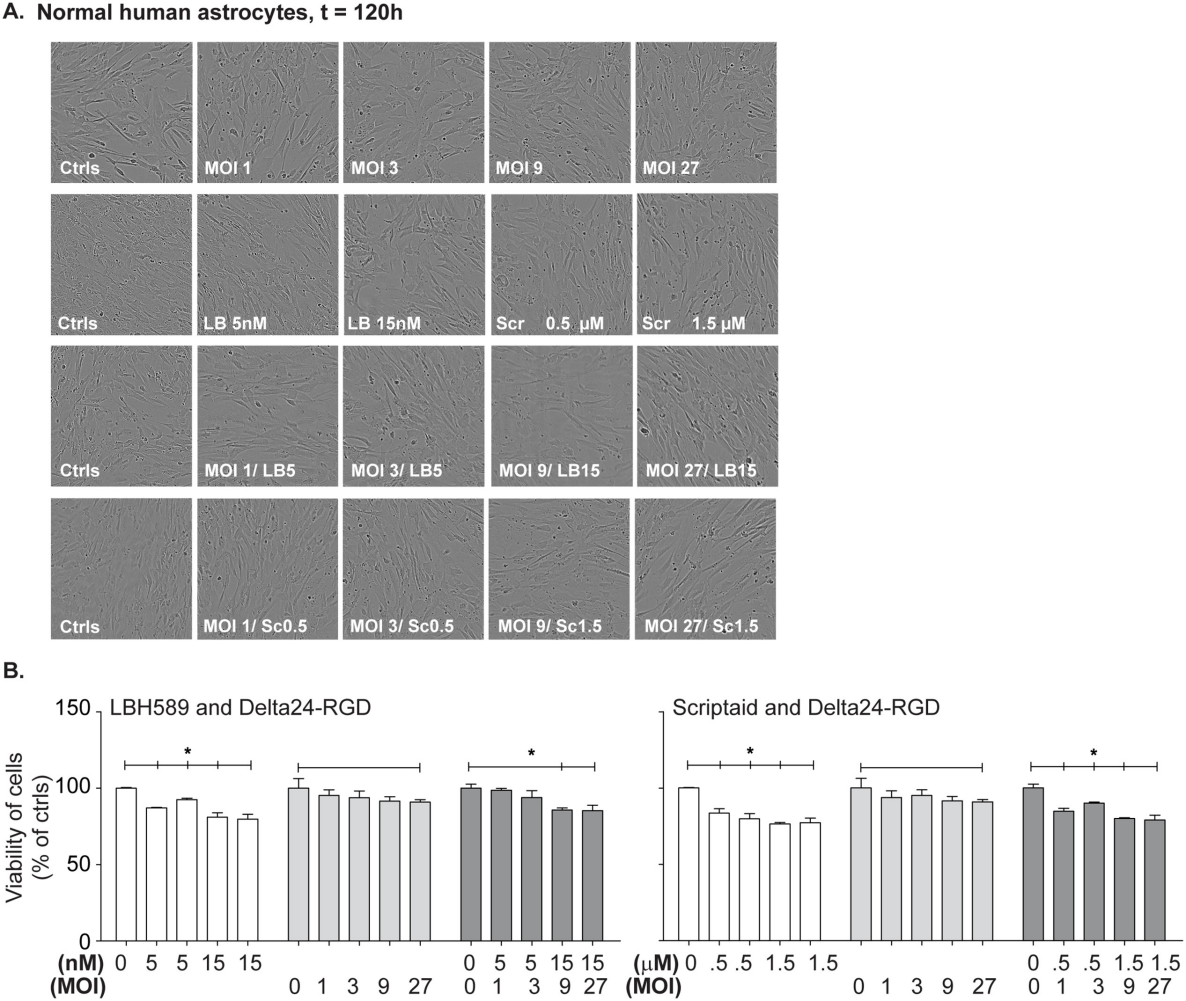
The effects of HDACi/Delta24-RGD on normal human astrocytes. (A) Normal human astrocytes were concomitantly treated with a dose range of HDACi and Delta24-RGD and monitored for morphological changes using the IncuCyte imaging system. Representative images at day five are depicted (10X magnification) for the tested conditions. (B) Viability of the cells was measured at day five post-treatment using the CellTiter-Glo assay, and the results are shown as percentage of non-treated control cells ± standard deviation. *Indicates significance at p<0.05 of the treated cells compared to the non-treated control cells.

In addition to the viability experiments on GSCs, we tested the drugs in combination with Delta24-RGD on normal human astrocytes to gain insight into toxicity of these regimens. The normal human astrocytes were treated with various concentrations of drugs, virus and the combination of both. Phase contrast images obtained at 120 hours and did not reveal any morphological changes as depicted in Fig 6. Moreover, cell growth confluence data derived from this time course experiment detected no significant effects of any of the tested treatment combinations (S3 Fig). Viability assessment by CellTiter-Glo assay showed very limited toxicity, with less than 20% reduction in cell viability (Fig 6). No additive effects of the combination treatments were detected for any of the tested concentrations.

## Discussion

In the current study we show that HDACi, in particular the novel pan-HDACi Scriptaid and LBH589 are effective sensitizers for the oncolytic virus Delta24-RGD in a subset of patient-derived GSC cultures. The limited toxicity to normal human astrocytes makes these drugs interesting candidates for further investigation for combination treatment with Delta24-RGD. These two HDACi have not been reported in combination with oncolytic virus therapy thus far. Notably, our data obtained on patient-derived GSCs, indicate that response to HDACi and Delta24-RGD combination treatment varies between the types of HDACi and between the different GSCs, with distinct responders and non-responders. Although we aimed at correlating the treatment efficacy to tumor subtype or profile by various methods, no clear relationships were observed between specific molecular markers and Scriptaid/Delta24-RGD or LBH589/Delta24-RGD responsiveness. An exception is the potential relationship between expression levels of Rb-pathway genes and LBH589/Delta24-RGD response, and αv integrin expression and Scriptaid/Delta24-RGD response. Larger patient-derived culture panels are required to substantiate this. Reports by others indicate that HDACi interact differentially with the activity of various oncolytic viruses: VPA was found to be an effective sensitizer of oncolytic herpes virus[24], adenovirus[25, 26] and vaccinia virus[27]. In contrast, VPA acted antagonistically with an oncolytic adenovirus.[28] In our previous study testing anti-epileptic drugs in combination with Delta24-RGD, the effects of VPA were very moderate and enhanced Delta24-RGD oncolysis only in a small subset of tumors.[8] Together, these reports underscore the variability in response with dependencies on tumor cell (sub)type, HDACi, and oncolytic virus strain.

In the present study, the two most effective HDACi, Scriptaid and LBH589, were further investigated in an additional panel of six GSCs. Over 50% of the total of fourteen distinct GSCs responded to Scriptaid-induced viral sensitization and 50% responded to LBH589-induced viral sensitization. Efficacy was related to HDACi-induced up-regulation of αvβ3 integrin expression and consequential increased infection efficiency of Delta24-RGD. Both HDACi asserted this effect in responding, but not in non-responding GSCs. Our data suggest that combined efficacy of HDACi and Delta24-RGD is at least partially determined by improved viral attachment and infection. The finding that responders have higher baseline gene expression levels of integrins than the non-responders may suggest that if gene expression is relatively high, these cells are able to further increase integrin production, whereas in non-responders, where gene production is already low or disturbed, this cannot be increased by the HDACi. VPA has previously been reported to alter integrin expression on the tumor cell surface.[29] Likewise, the HDACi TSA and sodium butyrate enhanced CAR levels and oncolytic adenoviral efficacy.[30, 31] In our study, CAR was not consistently up-regulated in the patient-derived GSCs, which further supports that HDACi effects are cell type and time-dependent. The αvβ3 integrin expression can be partially regulated by the transcription factor Sp1 [32], which was previously reported to be induced by HDACi.[33].

The fact that the increased viral infection and late viral gene expression in our study, did not translate to increased viral yield, suggests that combination therapy supports early cell killing instead of viral replication. We hypothesize that the activation of cell death mechanisms by the HDACi interferes with the final stage of the viral lytic cycle, and hampers the assembly of new viral particles, a process that requires active cellular machineries. Indeed, we found that there were relatively more dead cells at 48 and 96 hours, as apoptosis, autophagy and necrosis markers were elevated compared to the monotherapies. Apoptosis[5], autophagy[6] and necrosis-like cell death[5] are important mechanisms in Delta24-RGD adenoviral oncolysis.[6, 34] Autophagy and necrosis play a major role in the immunogenic cell death.[35] With regard to the effects of HDACi, not only cell death is important but also the reported induction of the cell cycle arrest proteins p21 and p27 [36], which induce cell cycle inhibition and dormancy in cellular replication machineries. These effects of HDACi can explain the limited effects, despite the increased infection, on nett viral replication and production.

We found that multiple mechanisms occur simultaneously in combination therapy. First of all, early (0–60 hours) apoptosis plays a role specifically in Scriptaid combination treatment. Secondly, autophagy plays a role for both HDACi (72 hours) as was observed by p62 consumption and LC3B conversion, as well as by phospho-p70S6K inhibition. Third, the LDH induction by degraded cells cannot be explained only by apoptosis and autophagy, which suggest that other cell death mechanisms are active, including necrosis. The observed effects of the HDACi, whether that is Scriptaid enhancing apoptosis, autophagy and necrosis, or LBH589 supporting autophagy and necrosis, can offer an advantage in boosting therapeutic efficacy by multiple mechanisms simultaneously. Moreover, enhancement of immunogenic cell death may offer the additional value of boosting the virus-induced anti-tumor immune response, known to play a key role in oncolytic viral therapy *in vivo*.[37–39] HDACi have been reported to induce antigen presentation and MHC-I induction, which could potentially contribute to T-cell activation and *in vivo* efficacy of the combination treatment.[40] Therefore, further studies into these aspects of HDACi/Delta24-RGD treatment are warranted.

## Conclusions

The novel pan-HDACi Scriptaid and LBH589 in combination with Delta24-RGD exert enhanced anti-tumor activity in patient-derived GSCs. The HDACi induced slight up-regulation of cell surface integrins, facilitating adenoviral entry and leading to increased levels of viral gene expression. Moreover, the HDACi (differentially) induced cell death pathways in the GSCs, thereby accelerating the virus-induced killing of the infected cells but slightly hampering the production of progeny virus. The concerted action of these two treatment modalities leads to improved anti-tumor efficacy in patient-derived GSCs and shows very limited toxicity in normal human astrocytes. Taken together, Scriptaid and LBH589 offer opportunities potential candidates for future Delta24-RGD combination studies.

## Supporting Information

S1 Fig Six additional GSC cultures were tested for combination effects of HDACi and Delta24-RGD. Results are shown for one dose of the drugs Scriptaid and LBH589 and one dose of the oncolytic virus, as indicated. Response was defined as an enhancement factor >1 (Table 1), which was significantly different from both single agents (p<0.05). The red bars indicate resistant GSCs to combined treatment whereas the blue bars indicate sensitive GSCs to combined treatment. The results are displayed by the mean viability percentage compared to non-treated controls with the standard deviations. *Indicates significance of combination treatment compared to drug or Delta24-RGD alone, p<0.05.
(TIFF)

S2 Fig The cells of the two responsive GSCs GS102 and GS289, were treated with LBH589 and Scriptaid as indicated. After 6 hours of treatment, the cells were harvested and the integrin αvβ3 (left) and CAR (right) levels were determined by flow cytometry analysis.
(TIFF)

S3 Fig Confluence of the cells was measured at day five post-treatment by the IncuCyte imaging system and software, and is presented as percentage of non-treated control cells ± standard deviation. *Indicates significance at p<0.05 of the treated cells compared to the non-treated control cells.
(TIFF)

S1 Table The correlation data of responses to HDACi/Delta24-RGD and molecular characteristics of the patient-derived GSC cultures, including *TP53* mutation status, HDAC gene expression, Rb-pathway gene expression, and integrin gene expression levels. (XLSX)
