# Supplementary figures and images for: The HDAC Inhibitors Scriptaid and LBH589 Combined with the Oncolytic Virus Delta24-RGD Exert Enhanced Anti-Tumor Efficacy in Patient-Derived Glioblastoma Cells
Source: PLoS One. 2015 May 18;10(5):e0127058. doi: 10.1371/journal.pone.0127058 (PMC4436250; doi:10.1371/journal.pone.0127058)

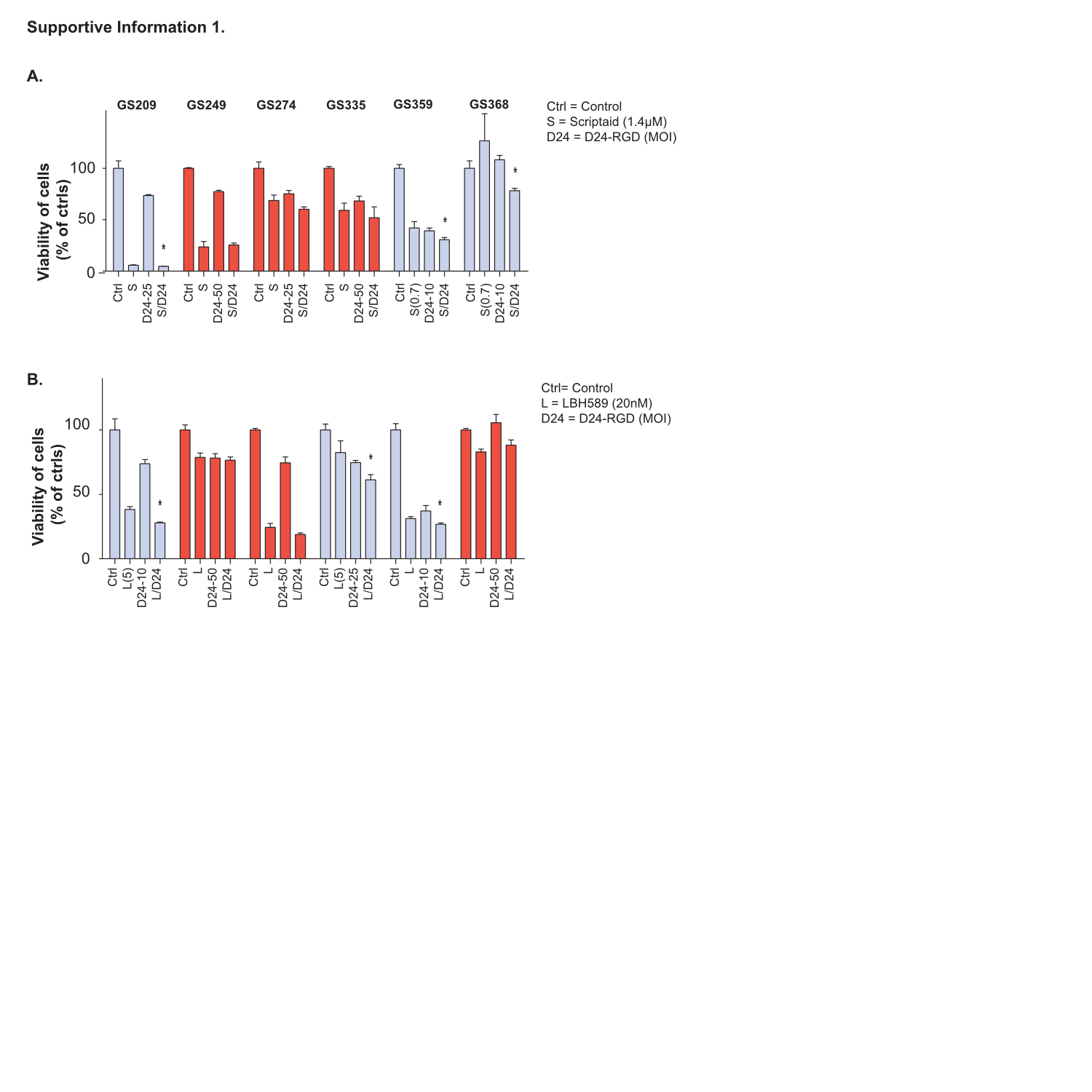

Supplement: S1 Fig — Results are shown for one dose of the drugs Scriptaid and LBH589 and one dose of the oncolytic virus, as indicated. Response was defined as an enhancement factor >1 (Table 1), which was significantly different from both single agents (p<0.05). The red bars indicate resistant GSCs to combined treatment whereas the blue bars indicate sensitive GSCs to combined treatment. The results are displayed by the mean viability percentage compared to non-treated controls with the standard deviations. *Indicates significance of combination treatment compared to drug or Delta24-RGD alone, p<0.05. (TIFF) [file pone.0127058.s001.tiff]

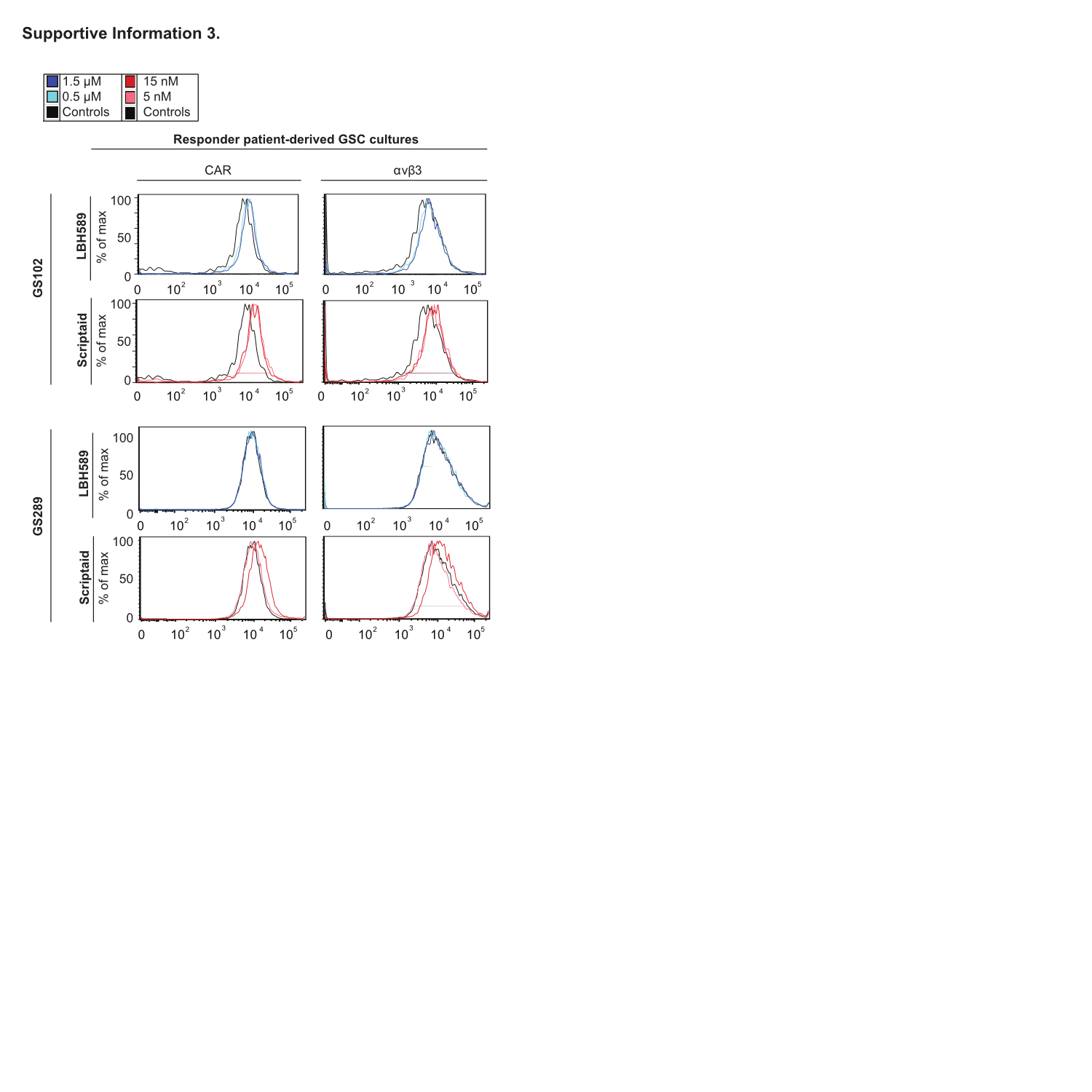

Supplement: S2 Fig — After 6 hours of treatment, the cells were harvested and the integrin αvβ3 (left) and CAR (right) levels were determined by flow cytometry analysis. (TIFF) [file pone.0127058.s002.tiff]

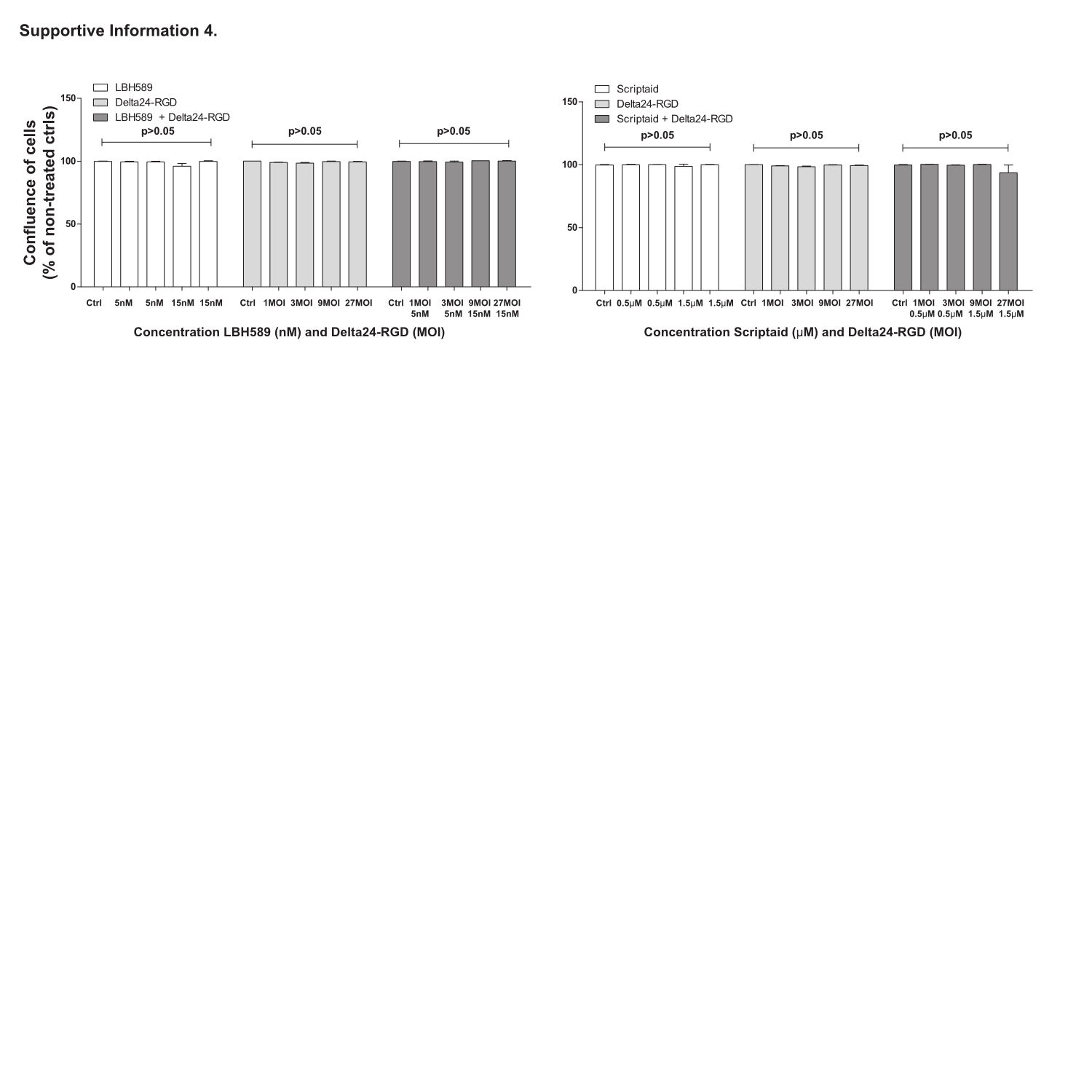

Supplement: S3 Fig — *Indicates significance at p<0.05 of the treated cells compared to the non-treated control cells. (TIFF) [file pone.0127058.s003.tiff]
